# Nutrition, environment and cardiovascular health (NESCAV): protocol of an inter-regional cross-sectional study

**DOI:** 10.1186/1471-2458-10-698

**Published:** 2010-11-15

**Authors:** Ala'a Alkerwi, Michèle Guillaume, Faiez Zannad, Ulrich Laufs, Marie-Lise Lair

**Affiliations:** 1 Centre de Recherche Public Santé, Centre d'Etudes en Santé, Grand-Duchy of Luxembourg; 2 School of Public Health, University of Liège, Belgium; 3 Centre Hospitalier Universitaire, Hypertension Unit, Département des maladies cardiovasculaires, Nancy, France; 4 University of Saarland, Germany

## Abstract

**Background:**

Despite the remarkable technological progress in health care and treatment, cardiovascular disease remains the leading cause of premature death, prolonged hospitalization and disability in most European countries. In the population of the Greater Region (Grand-Duchy of Luxembourg, Wallonia in Belgium, and Lorraine in France), the prevalence of cardiovascular risk factors and disease is among the highest in Europe, warranting the need for a better understanding of factors contributing to this pattern. In this context, the cross-border "Nutrition, Environment and Cardiovascular Health-NESCAV" project is initiated by an inter-regional multi-disciplinary consortium and supported by the INTERREG IV A program "Greater Region", 2007-2013, to fight synergically and harmoniously against this major public health problem.

**Methods/design:**

The objectives of the three-year planned project are to assess, in a representative sample of 3000 randomly selected individuals living at the Greater Region, 1) the cardiovascular health and risk profile, 2) the association between the dietary habits and the cardiovascular risk, 3) the association of occupational and environmental pollution markers with the cardiovascular risk, 4) the knowledge, awareness and level of control of cardiovascular risk factors, 5) the potential gaps in the current primary prevention, and finally, to address evidence-based recommendations enabling the development of inter-regional guidance to help policy-makers and health care workers for the prevention of cardiovascular disease.

**Discussion:**

The findings will provide tools that may enable the Greater Region's decision-makers and health professionals to implement targeted and cost-effective prevention strategies.

## Background

Despite the remarkable technological progress in health care and treatment, cardiovascular disease (CVD) remains the leading cause of premature death in most European populations [1]. It accounts for over 4 million deaths yearly, i.e., nearly half (49%) of all European mortality, but with striking geographical variations[2]. CVD represents an important source of prolonged hospitalization, and disability, contributing therefore to escalate the costs to health care systems[3].

In the frame of the European inter-regional program, Interreg III A 2003-2006, covering the Greater Region located in the heart of Europe, a cross-border study was carried out to explore the availability of information regarding the cardiovascular health indicators, such as the mortality, morbidity, and risk factors among the population. The study associated three neighbouring regions; Grand-Duchy of Luxembourg, Wallonia in Belgium, and Lorraine in France. While the cardiovascular mortality data were merely available for the period of 1987 to 1997, the comparative study revealed a substantial data shortage regarding cardiovascular morbidity and lifestyle-related risk factors, particularly in Luxembourg and Wallonia. The huge inter-regional discrepancies in the health information systems were not only related to heterogeneous methods and tools used for data collection, but also to total absence of data as regards to important potentially modifiable risk factors, for instance, dietary habits and physical activity[4].

Over the last decade, a growing body of epidemiological and clinical evidence has raised the potential deleterious effects of air pollution on health and its relation to heart disease and stroke [5]. An insight about the occupational and environmental pollution and its relation to cardiovascular risk was almost deficient in the three regions.

The inter-regional information gaps and data inconsistency precluded relevant cardiovascular health indicator's comparison[4]. From prevention point of view, this outcome made epidemiological cardiovascular health monitoring, particularly with regard to dietary and environmental factors, as one of the major topics for the Greater Region's public health authorities.

Cardiovascular disease prevention became a crucial contemporary public health challenge. The multi-factorial etiology of cardiovascular disease implies multi-facetted intervention program, to modify individual cardiovascular risk factors with diet, exercise, weight management, complete smoking cessation, and avoidance of environmental pollution [6].

Against this background, the idea of cross-border cardiovascular health monitoring was born in 2005. The outline of a standard protocol was conceived by the inter-regional committee which consisted of partners from the three regions, with various skills in the fields of public health, epidemiology, cardiology, nutrition and environment.

Under the auspices of INTERREG IV A program, 2007-2013, the Public Health Research Centre in Luxembourg (CRP-santé) initiated and coordinated the implementation of a complementary cross-border project; entitled "Nutrition, Environment and Cardiovascular Health NESCAV". This project includes the same 3 neighbour regions: Grand-Duchy of Luxembourg, Wallonia, Lorraine, in addition to Saarland. Its main goals are to build up a standard instrument to evaluate cardiovascular health and risk factors profile and to identify the potential gaps in the current primary prevention approaches in order to develop effective inter-regional networks and partnerships for concerted global action across the Greater Region. The ultimate aim of the multi-disciplinary NESCAV project is to fight efficiently against this major public health challenge, presented by the cardiovascular risk.

### Objectives

Within the NESCAV project, and in a representative sample of the population living in the Greater Region, 6 key objectives are developed: 1) describe and assess the cardiovascular health and risk profile, 2) explore the association between the dietary habits and the cardiovascular risk, 3) describe and assess the occupational and environmental pollution markers and examine their association with the cardiovascular risk, 4) assess the knowledge, awareness and level of control of cardiovascular risk factors (Hypertension, diabetes and dyslipidemia), 5) identify the potential gaps in the current primary prevention in order to improve the existing approaches and promote cross-border cardiovascular health, and 6) address evidence-based recommendations enabling the development of inter-regional guidance to help policy-makers and health care workers for the prevention of cardiovascular disease.

### Hypotheses

Although the NESCAV study is mainly descriptive, aimed at generating hypotheses, however, we formulate the following assumptions. First, there are significant inter-regional population disparities, with regard to cardiovascular health and risk profile. Second, the dietary habits are remarkably different across the population of the Greater Region, which may explain, in part, the observed cardiovascular risk discrepancy. Third, the populations of the Greater Region are exposed to different levels of occupational and environmental pollution, which potentially influence their cardiovascular health and risk profile. Fourth, the knowledge, perception and control of cardiovascular risk are varied significantly across the population living in the Greater Region. Fifth, the current preventive interventions, carried out in this Greater Region did not target the real population at risk. Sixth, the self-declared level of tobacco and alcohol consumption is significantly lower than the objective confirmation via biomarkers.

## Methods/Design

**Figure 1:**
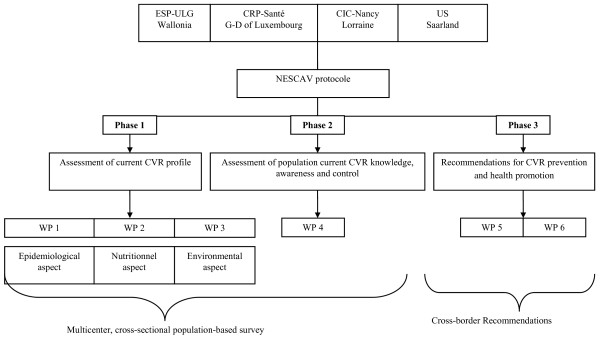
**Overview of NESCAV project structure**. ESP-ULG: School of Public Health-University of Liège; CRP-Santé: Public Health Research Centre; CIC: Centre of Clinical Investigation; US: University of Saarland; WP: Work package, NESCAV: Nutrition, Environment and Cardiovascular Health; CVR: cardiovascular risk.

The three-year planned project, began in 1^st ^January 2009, comprises three principal phases, each with stand-alone objectives and working packages (WP) (Figure 1):

### I. Asessment of current cardiovascular health and risk profile

This phase of the project enfolds 3 interrelated WPs that aim to achieve the first 3 above-mentioned objectives. It plans to carry out, for the fist time in this European zone, a cross-sectional, population-based survey, by using a standardised method of recruitment, and validated data collection tools. In line with the step-wise strategy of the WHO[7], this approach enables a relevant inter-regional comparison, in terms of cardiovascular health and lifestyle-related risk factors. From a public health and research perspective, this cross-border survey is intended to be repeated at regular intervals to monitor the evolution. The cardiovascular health status is assessed by means of a self-administered questionnaire, clinical and anthropometric measurements, as well as by blood, urine and hair examinations. The potentially modifiable and treatable risk factors include smoking, alcohol consumption, dietary habits, physical activity, hypertension, dyslipidemia, diabetes mellitus, obesity and environmental pollution. This cross-border survey attempts to demonstrate the potential contribution of the demographic, socio-economic, behavioural, biological and environmental factors to the cardiovascular risk, in a representative sample of non-institutionalized participants, aged 18-69 years residing in the Greater Region. The minimal necessary representative sample size was calculated to 3000 subjects to ensure statistical power[8] for examining the various cardiovascular risk factors of interest, i.e. to ensure a statistical precision of at least 2% for the estimation of the prevalence of the risk factors at the 95% confidence level. The distribution of selected subjects in each region is proportional to their distribution in the source population (adult population of 18 to 69 years residents in the Greater region). Pregnant women, people living in institutions, subjects outside the age range 18-69 years and those deceased before recruitment are excluded.

Given the multi-linguistic nature of the population residing in this zone, notably for the G-D of Luxembourg, the self-administered questionnaire is translated from French into the three other most used languages; German, English and Portuguese. To ensure the validity, the questionnaire is after that backward translated into French [9].

For field work, survey staffs are trained regionally to perform the anthropometric measurements and to help and check the answers with the participants. For each region, strict control measures are applied to ensure quality throughout the conduct of the cross-border survey, namely, sample selection, operational data-collection, data processing and reporting. Although, subject's recruitment and field data collection are organised at regional level, a centralized databank is constituted to introduce inter-regional data, with an on-line access to the 4 partners (Capture system^®^). The project's implementation and conduct is governed by an official agreement between the four partners.

First, 2^nd ^and 3^rd ^WPs focus on the epidemiological, nutritional and environmental aspects of the cardiovascular risk profile, respectively. The food intake frequencies and eating habits are assessed by a semi-quantitative food frequency questionnaire (FFQ), developed and adapted to regional-cultural and linguistic particularities by the University of Liège in Wallonia. The FFQ aims to assess the dietary intake, by asking the participants to report the frequency of consumption and portion size of approximately 134-line items over the last three months. Each item is defined by a series of foods or beverages which are categorized into 9 major food groups: starchy food, fruits, cooked and raw vegetables, meat-poultry-fish-eggs, prepared dishes, diary products, fats, divers and, drinks (alcoholic and non-alcoholic). The Participants reported the frequency of consumption of each food group on the basis of 6 levels of frequencies, ranging from rarely or never or <1 time a day to 2 times or more per day. Standard serving sizes and food models based on a photographic manual, validated by the SU.VI.MAX study[10], are provided as a reference for estimation of food consumed portions.

The assessment of cross-border occupational and environmental pollution and its relationship to cardiovascular risk profile is achieved by hair specimens' examination for specific biomarkers, namely the polycyclic aromatic hydrocarbons (PAHs) and pesticides, in addition to information collected by the self-administered questionnaire, such as the area of residence, type of job, smoking and alcohol consumption.

To meet the urgent needs of Luxembourg's public health authorities, for relevant baseline information about the population cardiovascular health status, WP 1, 2 &3 were carried out earlier in Luxembourg, under the auspices of the Ministry of Health and co-financed by the Ministry of Research. This part of NESCAV project corresponds to the national study, entitled Cardiovascular Risk Factors Observation in Luxembourg "ORISCAV-LUX". Detailed methodology was published elsewhere[11,12]. The foregoing achievement of this part helped as a learning example of feasibility for the other regions. The data collection of this part of NESCAV project is currently ongoing in the others regions.

### II. Assessment of current population knowledge, perception and control of cardiovascular risk factors

The population knowledge, perception and awareness of the impact of cardiovascular risk factors on their global health, are essential components of behavioral changes and lifestyle modification[13-15]. Likewise, the patient's compliance, self-cardiovascular risk management and the level of therapeutic control contribute to decrease the burden. The lack of knowledge, misconceptions, and the poor level of risk factor control are attributed to ineffective prevention programs and to confusing educative messages addressed to inappropriate groups of the population[16]. In order to develop effective strategies, tackling "high-risk group" or "population-targeted" strategies, it is crucial to understand how different populations view and experience cardiovascular health and diseases [17]. However, little is known about these issues among the residents of the Greater Region.

This part of the NESCAV project, corresponding to the 4^th ^WP, consists of three sub-steps; questionnaire development, testing-retesting and validation on a sample of 41 subjects resident in the Greater Region. Similarly, the questionnaire is backward translated into 4 languages and integrated in the general 1^st ^phase cross-border population-based NESCAV survey questionnaire. This part provides a unique opportunity to examine the knowledge, awareness, perception and self-control of 3 major cardiovascular risk factors-associated pathologies (diabetes, hypertension and dyslipidemia) among the general Greater Region's population. Standard data on current lifestyles-related cardiovascular risk factor and their management are crucial, not only to establish an objective view of the present population's health status but also to identify the potential gaps in health care or primary prevention approaches, hence to set-up specific target interventions tailored to real needs of the population. Given the magnitude of the problem and the financial burden of CVD, improvements in the quality of primary prevention will lead to substantial improvement in health care outcomes [18].

### III. Evidence-based recommendations for cardiovascular prevention and health promotion

It is well-established that a high proportion of CVD can be prevented and controlled by lifestyle modifications[19-21]. Several prospective observational studies showed that cross-cultural health disparities and low socio-economic position, measured as education level, income, or occupational class were associated with increased risk for type 2 diabetes[22] and coronary heart disease[23,24]. Evidence-based recommendations tailored to target group at risk are therefore fundamental to fight efficiently against CVD and mortality.

In most of European countries, diverse prevention programs and health promotion campaigns, aiming to endorse population and individual healthy lifestyles, were planned and organized, to various extents. Ideally, this should be a systematic process following a prior thorough needs assessment, data analysis and hence objective selection of prevention goals. A program best suited for target population is essential to implement credible and sustainable prevention. However, evidence-based CVD prevention programs are currently scarce and their impacts or effectiveness on the population at risk are rarely evaluated.

In this context, the 3^rd ^phase of the NESCAV project focuses on bridging the gaps potentially identified in the initial parts of the project. It is composed of two WPs: WP5 which seeks to spot the current primary prevention approaches and educative messages in each region, and WP6 which aims to suggest evidence-based recommendations appropriate to Greater Region's population needs. In the light of findings obtained from the first 4 WP, this final part of the project aims to develop inter-regional networks and stakeholders' partnerships for concerted actions of prevention across the Greater Region to fight efficiently against cardiovascular disease.

#### Ethical aspects

The protocol of the first 4 WPs of the study was approved by the following institutional review boards: *Comité National d'Ethique de Recherche *(Grand-Duchy of Luxembourg), *Comité de Protection des Personnes Est-III *(Lorraine), *Comité d'Ethique Hospitalo-Facultaire Universitaire de Liège (Wallonia) and Ethik-Kommission Ärztekammer des Saarlandes *(Saarland).

In each region, an informed written consent form is handed to each participant at his/her attendance visit, to be signed prior to inclusion. In Grand-duchy of Luxembourg, alike to the questionnaire, the consent was provided in 4 languages (French, English, German and Portuguese).

## Discussion

Based on the findings of cross-border NESCAV survey, it will be possible to establish for the fist time an objective panorama of cardiovascular health status of the Greater Region's population. However, the challenge is not merely to monitor the cardiovascular risk factors, but to evaluate the current risk perception and management from population's standpoint, in order to bridge the knowledge-practice gaps and to suggest evidence-based recommendations. The findings will enable the decision-makers and health professionals to implement effective and target prevention strategies. At medium to long term, the outcome may have an impact on the global health of the Greater Region population, in terms of prevention and health care policies as well as on the economy.

## Competing interests

The authors declare that they have no competing interests.

## Authors' contributions

AA coordinated the 1^st ^phase of the project for Luxembourg region, drafted the present manuscript and participates to scientific decisions at interregional level. M-LL wrote the NESCAV project protocol in collaboration with the partners, in the light of final report of the previous project INTERREG III, coordinated the work packages at inter-regional level, and supervised all the cross-border project process and data analysis of results. M-LL, MG, FZ, UL are the NESCAV project leaders of Luxembourg, Wallonia, Lorraine and Saarland, respectively. All authors commented on the design of the NESCAV protocol and on drafts of the manuscript, and all approved the final version.

## Pre-publication history

The pre-publication history for this paper can be accessed here:

http://www.biomedcentral.com/1471-2458/10/698/prepub
